# No evidence for a common blood microbiome based on a population study of 9,770 healthy humans

**DOI:** 10.1038/s41564-023-01350-w

**Published:** 2023-03-30

**Authors:** Cedric C. S. Tan, Karrie K. K. Ko, Hui Chen, Jianjun Liu, Marie Loh, SG10K_Health Consortium, Minghao Chia, Niranjan Nagarajan

**Affiliations:** 1 Genome Institute of Singapore (GIS), Agency for Science, Technology and Research (A*STAR), Singapore, Republic of Singapore; 2 UCL Genetics Institute, University College London, London, UK; 3 Department of Microbiology, Singapore General Hospital, Singapore, Republic of Singapore; 4 Department of Molecular Pathology, Singapore General Hospital, Singapore, Republic of Singapore; 5 Yong Loo Lin School of Medicine, National University of Singapore, Singapore, Republic of Singapore; 6 Population and Global Health, Lee Kong Chian School of Medicine, Nanyang Technological University, Singapore, Republic of Singapore; 7 Department of Epidemiology and Biostatistics, Imperial College London, South Kensington, London, UK; 8 National Skin Centre, Singapore, Republic of Singapore

**Keywords:** Microbiome, High-throughput screening, Metagenomics

## Abstract

Human blood is conventionally considered sterile but recent studies suggest the presence of a blood microbiome in healthy individuals. Here we characterized the DNA signatures of microbes in the blood of 9,770 healthy individuals using sequencing data from multiple cohorts. After filtering for contaminants, we identified 117 microbial species in blood, some of which had DNA signatures of microbial replication. They were primarily commensals associated with the gut (*n* = 40), mouth (*n* = 32) and genitourinary tract (*n* = 18), and were distinct from pathogens detected in hospital blood cultures. No species were detected in 84% of individuals, while the remainder only had a median of one species. Less than 5% of individuals shared the same species, no co-occurrence patterns between different species were observed and no associations between host phenotypes and microbes were found. Overall, these results do not support the hypothesis of a consistent core microbiome endogenous to human blood. Rather, our findings support the transient and sporadic translocation of commensal microbes from other body sites into the bloodstream.

## Abstract (web summary)

A large population study indicates that there is no core blood microbiome in healthy humans.

## Main

In recent years, there has been considerable interest regarding the existence of a microbiome in the blood of healthy individuals, and its links to health and disease. Human blood is traditionally considered a sterile environment, where the occasional entry and proliferation of pathogens in blood can trigger a dysregulated host response, resulting in severe clinical sequelae such as sepsis, septic shock or death^1^. Additionally, asymptomatic transient bacteraemia (that is, bacterial presence in blood) in blood donors is known to be a major cause of transfusion-related sepsis^2^. Recent studies have suggested the presence of multiple microbial species circulating in healthy human blood^3–7^ (reviewed in ref. ^8^). However, most of these studies were either done in relatively small cohorts or lacked rigorous checks to distinguish true biological measurements from different sources of contamination^8^. As such, the concept of a microbial community in healthy human blood remains controversial. We analysed blood DNA sequencing data from a population study of healthy individuals, comprising multiple cohorts processed by different laboratories with varied sequencing kits. By leveraging the large dataset (*n* = 9,770) complete with batch information in our systematic analyses for potential contaminants, we investigated whether a blood microbiome truly exists in the general population.

For meaningful discourse, it is useful to formalize what a hypothetical ‘blood microbiome’ entails. The ‘microbiome’ should refer to a community of microbes that interact with each other and with the environment in their ecological niche^9^. Therefore, in a blood microbiome, microbes should exhibit community structures indicated by co-occurrence or mutual exclusion of species^10^ as seen in the microbiomes of other sites such as the gut^11^ or mouth^12^. Furthermore, we may expect the presence of core microbial species, which can be defined as species that are frequently observed and shared across individuals^13,14^, such as *Staphylococcus epidermidis* on human skin^15^. Taxa that are found in a substantial fraction of samples from distinct individuals (that is, with high prevalence) may be considered ‘core’. The prevalence threshold for defining core taxa is arbitrary, with previous microbiome studies using values ranging from 30–100% and many opting for 100%^14^. Regardless, identifying core microbes in blood would form the basis for associating microbiome changes with human health.

Existing evidence for a blood microbiome in healthy individuals comes from both culture-based^3,4^ and culture-independent^5–7^ approaches. The former involves blood culture experiments, while the latter includes the following molecular methods: 16S ribosomal RNA (rRNA) quantitative polymerase chain reaction (qPCR), 16S rRNA amplicon sequencing and/or shotgun sequencing of RNA or DNA. Depending on the study design, these results should be interpreted with caution due to several methodological and technical limitations including small sample sizes, limited taxonomic resolution, difficulties in distinguishing cell-free microbial DNA from live microbial cells and the ubiquity of environmental contamination^8,16–19^. In particular, microbial DNA contaminants introduced during sample collection and processing must be accounted for to characterize the blood microbiome. Contaminating microbial cells can also be introduced due to poor aseptic technique or insufficient disinfection of the skin puncture site^20^. Sequencing-based approaches are especially sensitive to microbial DNA contaminants native to laboratory reagent kits (that is, the ‘kitome’)^19^, exacerbated by the low microbial biomass and high host background in blood that increases the noise-to-signal ratio^17^. Few studies so far have provided a comprehensive profile of the breadth and prevalence of microbial species in blood in light of these challenges. Furthermore, several aspects of the ‘blood microbiome’ remain unclear: are the detected microbes endogenous to blood or translocated from other body sites? Is there a core set of microbes that circulates in human blood? Is there a microbial community whose structure and function could influence host health?

To address these questions, we performed presumably the largest-scale analysis of blood sequencing data so far, on the basis of DNA libraries for 9,770 healthy individuals from six distinct cohorts (Supplementary Table 1). We differentiated blood microbial DNA signatures from potential reagent contaminants and sequence analysis artefacts, leveraging the different reagent kits used to process each cohort. We detected 117 microbial species in the blood of these healthy individuals, most of which are commensals associated with the microbiomes of other body sites. Additionally, we identified DNA signatures of replicating bacteria in blood using coverage-based peak-to-trough ratio analyses^21,22^, providing a culture-independent survey that has not been done previously. Despite this, we found no evidence for microbial co-occurrence relationships, core species or associations with host phenotypes. These findings challenge the paradigm of a ‘blood microbiome’ and instead support a model whereby microbes from other body sites (for example, gut, mouth) sporadically translocate into the bloodstream of healthy individuals, albeit more commonly than previously assumed. Overall, our observations serve to establish a much needed baseline for the use of clinical metagenomics in investigating bloodstream infections.

## Results

### Inferring blood microbial DNA signatures with multicohort analysis

**Fig. 1:**
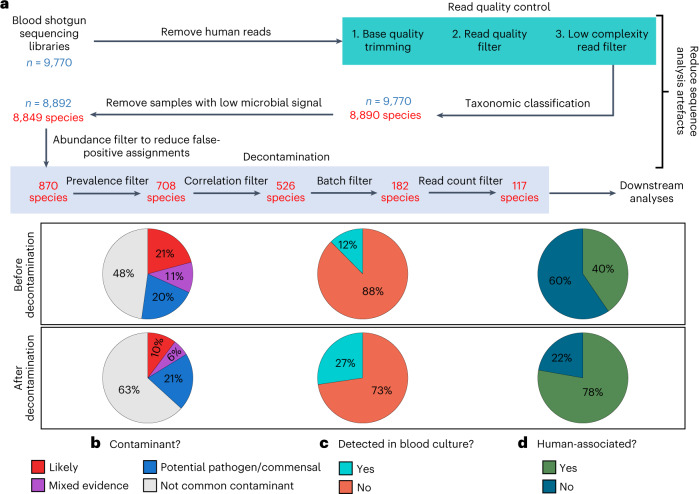
Robust identification of microbial DNA signatures in blood. **a**, Summary of pre-processing steps and filters applied to taxonomic profiles (*n* = 9,770 individuals) and the number of species retained after each filter. **b**–**d**, Pie charts showing the proportion of microbial species that are (**b**) common sequencing contaminants, (**c**) detected in blood culture records and (**d**) human-associated, before and after applying the decontamination filters. Source data

**Extended Data Fig. 1:**
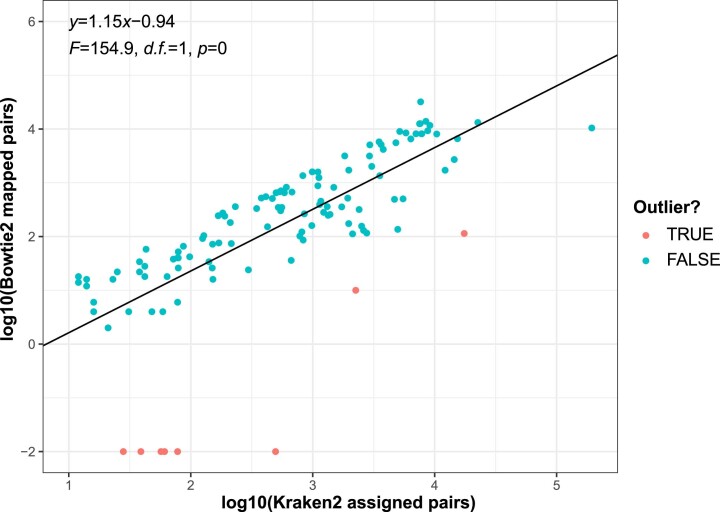
Strong linear relationship between the number of *Bowtie2* mapped and *Kraken2* assigned reads on the log10 scale. All data points (*n* = 122) are shown on the scatter plot. The linear regression line and associated parameter estimates annotated here were computed after removing outlier data points (in red). These outliers had studentised residuals >2 as computed from an initial linear regression including all data points. A two-sided *F*-test was used to determine if the slope parameter in the linear regression model differed from zero. Source data

Blood samples from healthy individuals typically contain low microbial biomass and high host DNA background^17^, making it difficult to discriminate between biologically relevant signals from artefactual ones. We first addressed artefacts arising during bioinformatic sequence analysis by performing stringent quality control on samples (Fig. 1a), comprising read-quality trimming and filtering, removing low-complexity sequences of ambiguous taxonomic origin, excluding human reads (Methods) and removing samples with low microbial reads (<100 read pairs). Following this, we obtained a species-level characterization of microbial DNA signatures in blood for most (*n* = 8,892) samples. To minimize false-positive taxonomic assigments, we discriminated between species that are likely present from those that could be misclassification artefacts using an abundance cut-off (Methods). We validated the reliability of the microbial species detected via ‘Kraken2’ (ref. ^23^) by aligning reads to their reference genomes, where a high coverage breadth delineated true positives from computational artefacts^24,25^. We further observed an excellent linear relationship between the number of Kraken2-assigned read pairs and the number of aligned read pairs on the log_10_ scale (slope = 1.15; two-sided *F*-test, *F* = 154, d.f. = 1, *P* < 0.001; Extended Data Fig. 1), suggesting that Kraken2 reliably identified taxa in blood. These findings collectively provide confidence that the microbial species detected in our blood sequencing libraries are not likely sequence analysis artefacts.

**Extended Data Fig. 2:**
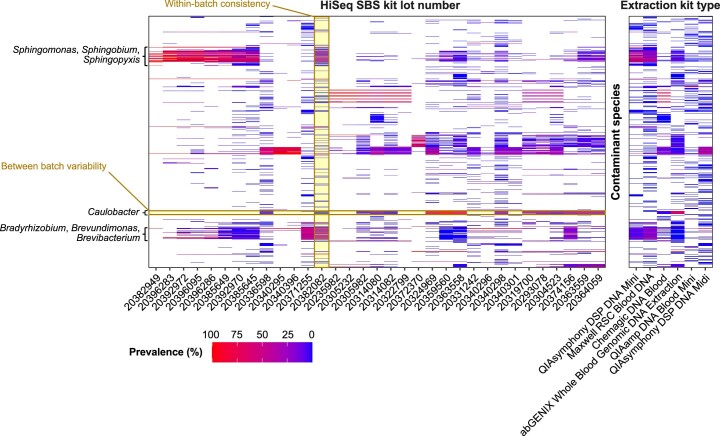
Between-batch variability and within-batch consistency of contamination signals. Heatmap of contaminant species prevalence stratified by the different lot numbers of the HiSeq SBS kit used for sequencing and by the different DNA extraction kits used to process the blood samples. Contaminant species are sorted by genus and notable genera known to be common contaminants are annotated on the figure. The prevalence of microbes varies greatly between the batches and kit types used (between-batch variability) and multiple species appear strongly correlated within a single batch (within-batch consistency). Source data

To address artefacts from reagent and handling contamination, we used a series of stringent decontamination filters (Fig. 1a and Methods). These filters are based on the observation that laboratory contaminants are often correlated with each other (within-batch consistency) and biased towards specific laboratory batches (between-batch variability; Extended Data Fig. 2)^26^. Similar analyses based on these patterns have been used previously and were found to be highly effective for the in silico identification of laboratory contaminants^27–29^. The identification of batch-specific contaminants in this study was aided by the availability of multiple large cohorts of healthy individuals (Supplementary Table 1) and corresponding rich batch information, including reagent kit types and lot numbers. After accounting for reagent and handling contaminants, we obtained a list of 117 microbial species that were detected in the whole blood samples of 8,892 individuals (Supplementary Table 2). These microbes spanned 56 genera comprising 110 bacteria, 5 viruses and 2 fungi.

**Extended Data Fig. 3:**
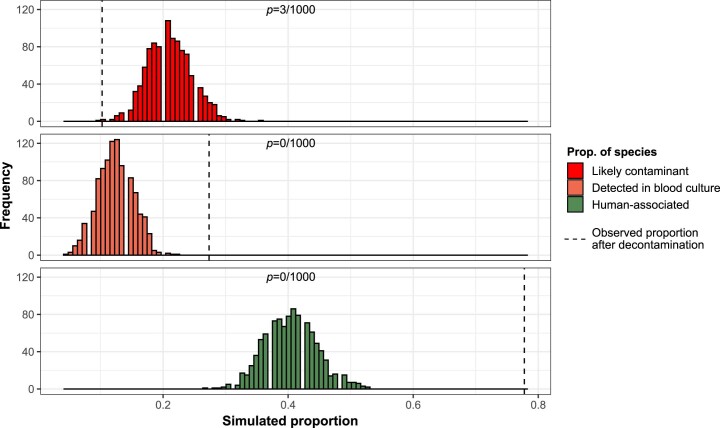
Decontamination filters significantly improve signal-to-noise ratio of microbial taxa retained. Null distributions for the proportions of species classified as not likely contaminants, detected in blood, or human-associated. To generate these null distributions, for each of 1000 iterations, we randomly selected 117 microbial species from the list of species before decontamination and classified them based on same procedure used to generate Fig. 1b–d. The observed proportions following the application of our decontamination filters are indicated by black dashed lines. P-values were calculated as the fraction of iterations where the species proportions were greater or equal to the observed proportions (one-sided test; no multiple-testing correction performed). Source data

To estimate the effectiveness of our filtering strategy in improving biological signal while reducing contamination noise, we examined the types of microbial species detected in our dataset before (870 species) and after (117 species) all filters were applied (Fig. 1b–d). First, the microbial species were cross-referenced against a published list of contaminant genera in sequencing data^19,30^. From this list, genera were either classified as likely contaminants, mixed-evidence (that is, both a pathogen and common contaminant) or potential pathogens/commensals. Following decontamination, the proportion of detected species that were classified as contaminants decreased from 21% to 10% (Fig. 1b). Next, the microbial species were compared against human blood culture records spanning more than a decade (2011–2021) from a tertiary hospital (Fig. 1c). The proportion of species that had been cultured from blood increased from 12% to 27% after decontamination, suggesting that our filtering procedures enriched for microbial species capable of invading the bloodstream. Finally, we compared the proportion of human-associated microbes before and after decontamination using a database describing the host range of pathogens^31^ (Fig. 1d). For species not found in this database, a systematic PubMed search (Methods) was performed to determine whether there was at least one past report of human infection. The proportion of human-associated species increased from 40% to 78% after decontamination, indicating that these species are more likely to be biologically relevant. Finally, we tested our results against the null hypotheses that the 117 microbial species retained after decontamination produced the same proportions of species classified as likely contaminants, human-associated, or that were detected in blood culture compared to species picked at random (Methods). Our decontamination filters significantly decreased the proportions of likely contaminants while increasing the proportions of human-associated species and those detected in blood cultures (all one-sided randomization tests *P* < 0.005; Extended Data Fig. 3). Overall, by using a set of contaminant-identification heuristics, our filters are sensitive and specific in retaining biologically relevant taxa while removing likely contaminants.

### Sporadic translocation of DNA from commensals in healthy blood

**Fig. 2:**
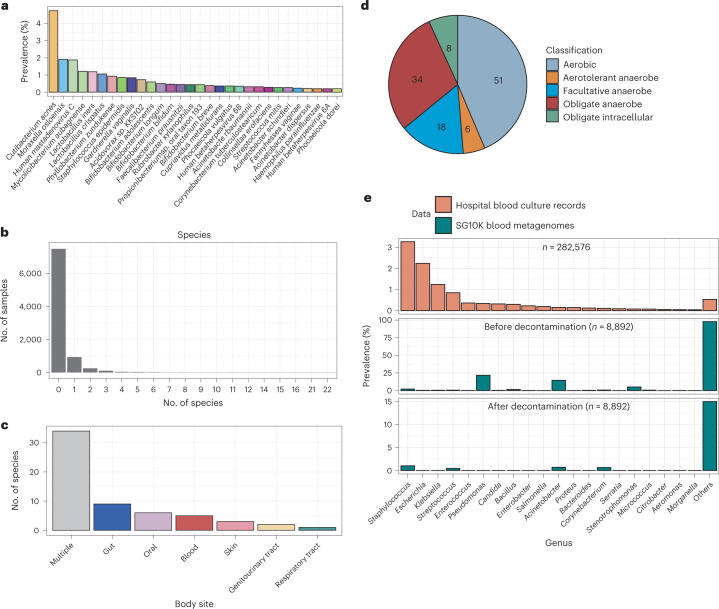
Microbial signatures in blood from healthy individuals. **a**, Bar chart showing the prevalence of the top 30 confidently detected microbial species in all 8,892 blood sequencing libraries. **b**, Histogram of the number of microbial species per sample. **c**, Bar chart of the human body sites the 117 confidently detected species are associated with, as determined using the Disbiome database^34^. Species are classified as ‘multiple’ if they are associated with more than one body site and classified otherwise if they are only associated with a single body site. **d**, Pie chart showing the microbiological classification of the 117 confidently detected species. **e**, Bar chart showing the prevalence of genera in blood culture records and in the blood sequencing libraries before and after decontamination. Source data

**Extended Data Fig. 4:**
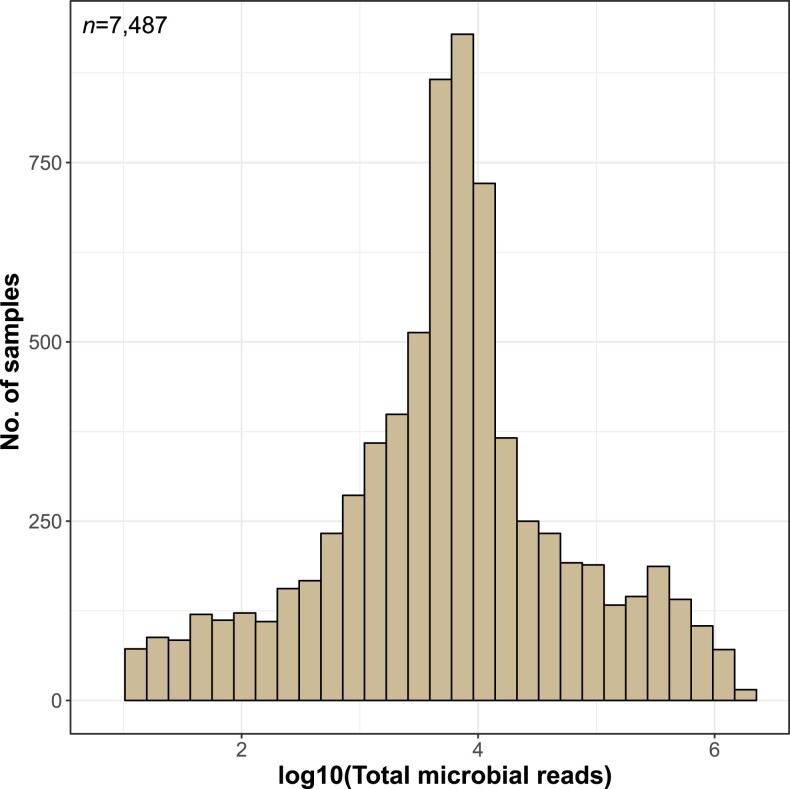
Distribution of total microbial reads for samples with no detected non-contaminant taxa. Total microbial read counts are equivalent to the number of reads classified as microbial after applying the abundance filter but before decontamination (see Methods, Fig. 1a). Source data

We next determined the fraction of healthy individuals for which microbes could be detected (that is, prevalence). The most prevalent microbial species, *Cutibacterium acnes*, was observed in 4.7% of individuals (Fig. 2a), suggesting that none of the 117 microbes were ‘core’ species across most healthy individuals. Additionally, we did not detect any microbial species in most (82%) of the samples after decontamination (Fig. 2b), whereas the remaining 18% had a median of only one microbial species per sample. Low microbial prevalence was not due to insufficient sequencing depth since there was a weak negative correlation between the number of confidently detected species and the total microbial read count per sample (Spearman’s *ρ* = −0.279, two-sided *t*-test, *P* < 0.001). Furthermore, some samples containing no microbial species had a microbial read count of up to ~2.1 million (median = 6,187 reads; distribution shown in Extended Data Fig. 4). Although a considerable number of reads were classified as microbial, they were all assigned to contaminant species. Our results suggest that the presence of microbes in the blood of healthy individuals is infrequent and sporadic.

Given past reports of bacterial translocation from the mouth^32^ or gut^33^ into blood, we asked whether the microbes we detected could have originated from various body sites. We assigned potential body site origins to our list of 117 blood microbes on the basis of microbe-to-body-site mappings extracted from the Disbiome database^34^. We found that many (*n* = 59; 50%) of the 117 species were human commensals associated with various body sites (Fig. 2c). While some of these species may be contaminants that have survived our stringent decontamination filters, this observation, together with their low prevalence, suggests that the DNA of many of these species may have transiently translocated from other organs rather than being endogenous to blood. A substantial proportion (*n* = 42; 36%) of the species were obligate anaerobes or obligate intracellular microbes atypical of skin-associated microbes that might have been introduced during phlebotomy^2^, indicating that they are not likely to be sampling artefacts (Fig. 2d). Overall, the diverse origins of the microbes detected in blood, together with their low prevalence across a healthy population, is consistent with sporadic translocation of commensals, or their DNA, into the bloodstream.

**Extended Data Fig. 5:**
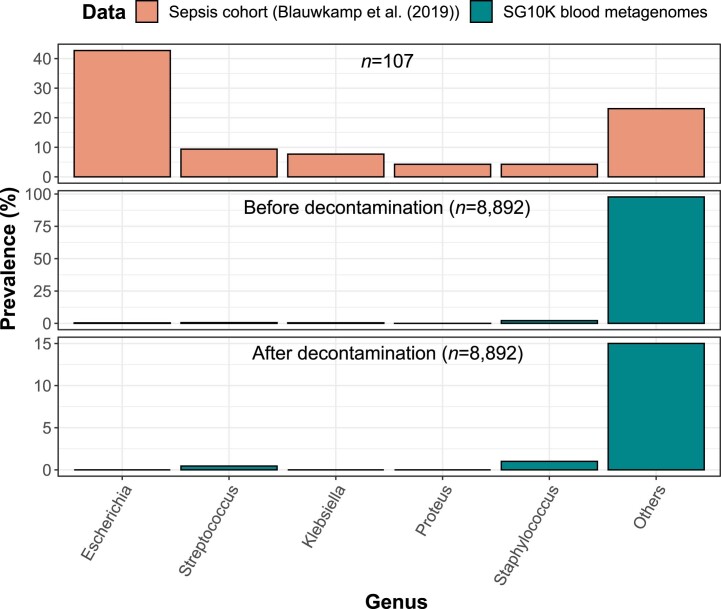
Microbial prevalence in sepsis patients differs from that in healthy individuals. Bar chart showing prevalence of genera detected in sepsis patients and in our blood sequencing libraries before and after decontamination. Blauwkamp et al. used shotgun sequencing of blood plasma collected from sepsis patients to determine the etiological agents involved and assessed the analytical sensitivity of this approach via multiple alternative culture-based and PCR-based detection methods. Source data

Bacteraemia is typically associated with a range of clinical sequelae from mild fevers to sepsis. We asked whether the common microbes identified in patients with bacteraemia were different from those in healthy individuals by comparing the prevalence of microbes in our dataset against observations from 11 years of hospital blood culture records. The prevalence of microbial genera from blood culture records clearly differed from that in our dataset, despite the overlap in detected taxa (Fig. 2e). For example, while *Staphylococcus*, *Escherichia* and *Klebsiella* were predominant in blood cultures, they were rare in our cohorts. We performed a similar comparison with a previous study^35^ that sequenced the blood of sepsis patients and found a similar difference in prevalence compared to our dataset (Extended Data Fig. 5), confirming that our observations were not due to differences in sequencing vs culture-based detection methods. A possible explanation for these differences could be the higher virulence of pathogens detected in the clinic, which are more likely to cause symptoms in individuals who would have been excluded during study recruitment. Conversely, if the microbial signatures in our dataset came from whole cells, these species might be better tolerated by the immune system in healthy individuals (for example, *Bifidobacterium* spp.^36^ and *Faecalibacterium prausnitizii*^37^ with immunomodulatory properties as gut commensals; Fig. 2a).

### Evidence of replicating microbes in blood sans community structure

**Fig. 3:**
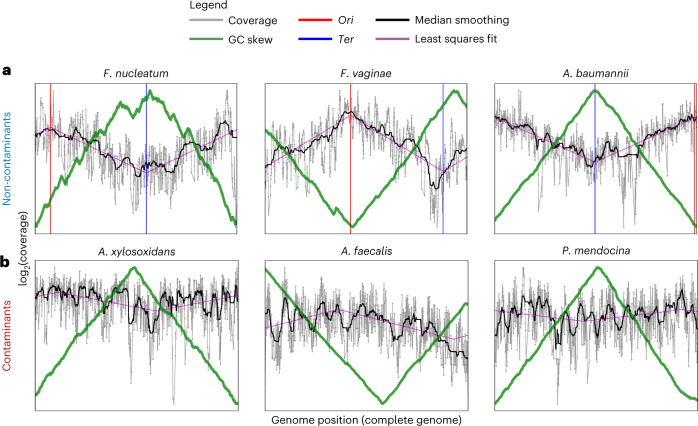
Evidence for replicating bacteria in blood samples from healthy individuals. **a**,**b**, Coverage plots of three representative (**a**) non-contaminant and (**b**) contaminant species. **a**, The sinusoidal shape of the coverage plots, characterized by higher depth of coverage nearer to the origin of replication (*Ori*) and lower coverage nearer to the terminus (*Ter*), is a signature of replicating bacterial cells. Source data

We asked whether blood microbial DNA signatures reflected the presence of viable microbial cells as opposed to circulating cell-free DNA. In contrast to previous approaches that used microbial cultures^3,38^, we looked for more broad-based evidence of live bacterial growth by applying replication rate analyses^21,22^ to our sequenced blood samples. In replicating bacteria, there should be increased coverage of DNA reads (that is, peak) nearer to the origin of replication (*Ori*) and decreased coverage (that is, trough) nearer to the terminus (*Ter*), leading to a coverage peak-to-trough ratio (PTR) > 1 (ref. ^22^). We found evidence for replication of 11 bacterial species out of the 20 that were sufficiently abundant to do this analysis (Table 1). The median-smoothed coverage plots of the replicating species all exhibited the sinusoidal coverage pattern (Fig. 3a, black pattern) characteristic of replicating bacterial cells^22^, contrasting with the even coverage patterns of three representative contaminants: *Achromobacter xylosoxidans*, *Pseudomonas mendocina* and *Alcaligenes faecalis* (Fig. 3b). The *Ori* and *Ter* positions determined using coverage biases largely corresponded with an orthogonal method based on the GC-skew^39^ of bacterial genomes, suggesting that the replication rate analyses are reliable. Additionally, all but one of these replicating species are present in hospital blood culture records and in previous reports of bacteraemia^40–49^ (Table 1), indicating their ability to replicate in human blood. Overall, beyond the detection of microbial DNA, we identified culture-independent molecular signatures for microbial replication in human blood.

**Table 1:** Summary statistics for samples where bacterial species were deemed to be replicating using *iRep*^21^ (that is, peak-to-trough ratio (PTR) > 1)

Subject ID	Species	Possible origin	Reported in blood	Read pairs assigned by Kraken2	Overall prevalence (%)	PTR
WHB4594	*Fusobacterium nucleatum*	• Genitourinary tract • Gut • Mouth	Yes	194,199	0.11	1.68
WHB9179	*Neisseria subflava*	• Gut • Mouth	Yes	15,385	0.16	1.51
WHB9179	*Haemophilus parainfluenzae*	• Gut • Mouth • Respiratory tract	Yes	12,183	0.2	1.17
WHB4035	*Fannyhessea vaginae*	• Genitourinary tract	Yes	10,395	0.24	1.88
WHB6459	*Staphylococcus epidermidis*	• Gut • Mouth • Respiratory tract • Skin	Yes	9,140	0.85	1.57
WHB10710	*Lactobacillus crispatus*	• Genitourinary tract • Gut • Mouth	Yes	7,799	1.06	1.57
0116–0053	*Acinetobacter baumannii*	• Mouth	Yes	7,673	0.31	1.9
WHB9179	*Neisseria flavescens*		Yes	3,787	0.06	1.38
WHB9978	*Rickettsia* sp. Tillamook 23		No	2,923	0.02	1.35
WHH1248	*Moraxella osloensis*		Yes	2,402	1.91	1.33
WHB9812	*Corynebacterium imitans*		Yes	1,976	0.02	1.59

**Fig. 4:**
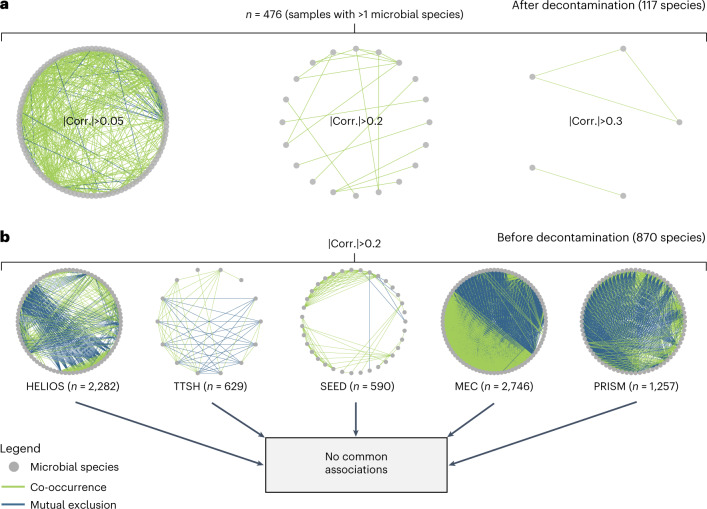
Microbial co-occurrence networks. **a**, SparCC^50^ co-occurrence networks computed for all samples with at least two microbial species following decontamination at different SparCC correlation thresholds (0.05, 0.2, 0.3). Only associations with a magnitude of SparCC correlation greater than the respective thresholds are retained. **b**, SparCC networks for individual cohorts at a correlation threshold of 0.2. No co-occurrence associations were retained after taking the intersection of edges between all cohort networks. In **a** and **b**, each node represents a single microbial species, and each edge a single association between a pair of microbial species. Edge thickness is scaled by the magnitude of correlation. The number of samples used to compute each network and the correlation thresholds used are annotated. Positive and negative SparCC correlations are indicated in green and blue, respectively. Source data

Given the DNA signatures of replicating bacteria, we investigated whether microbe–microbe interactions could be detected in healthy blood. We computed pairwise ‘SparCC’ correlations^50^ between species, where positive and negative values indicate co-occurrence and mutual exclusion, respectively. We visualized correlations of the 117 blood microbial species using network graphs (Fig. 4a). We could not detect strong community co-occurrence/mutual exclusion patterns, with most associations being weak (|correlation| < 0.05), and only 19 pairwise associations exceeding a correlation magnitude of 0.2 (Fig. 4a). To determine whether this was due to overly stringent decontamination, we generated independent network graphs for the five adult cohorts before decontamination and examined the co-occurrence/mutual exclusion associations shared across cohorts. We identified no associations common to all the network graphs (Fig. 4b), indicating that there were no consistent detectable microbial associations in blood that are typically seen in other microbiomes.

### No association between blood microbes and host phenotypes

**Extended Data Fig. 6:**
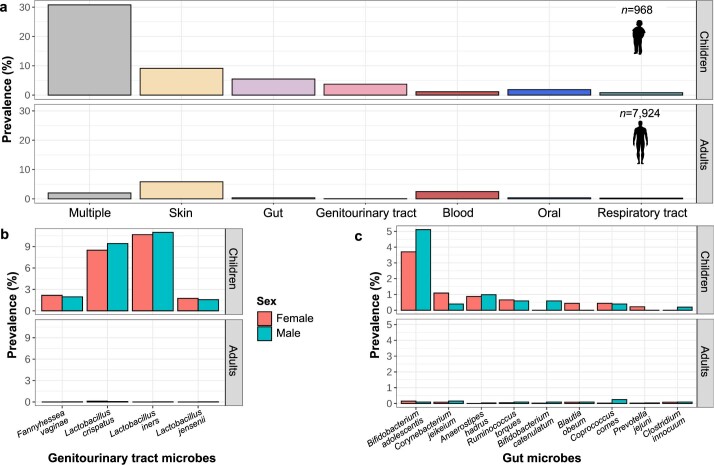
Higher prevalence of microbes in the blood of healthy children. (a) The disproportionately high prevalence of microbes in the children’s cohort GUSTO relative to the other adult cohorts. Silhouette icons were sourced from Adobe Stock with a standard license. Prevalence of (b) genitourinary tract-associated and (c) gut-associated microbes in children’s and adult cohorts, stratified by sex. Body site classifications were determined using the Disbiome database. Source data

Previous studies have used blood microbial DNA as disease biomarkers, demonstrating associations with cancer^30^, type II diabetes^51^ and periodontal disease^52^. Likewise, we investigated whether the presence of microbes was associated with host phenotypes in our dataset. We first examined whether microbes were detected more frequently in infants (GUSTO cohort) relative to adult cohorts, given that the still-developing immune systems of infants put them at a greater relative risk of infection^53^. GUSTO samples had a higher prevalence of microbes associated with most human body sites (Extended Data Fig. 6a). This was in part driven by genitourinary tract-associated microbes *Fannyhessea vaginae*, *Lactobacillus jensenii, Lactobacillus crispatus*, *Lactobacillus iners* and *Gardnerella vaginalis* (Extended Data Fig. 6b). Similarly, we found enrichment of gut-associated bacteria such as *Bifidobacterium* spp. in GUSTO (Extended Data Fig. 6c). These findings suggest that bacterial translocation may be more frequent in infants relative to adults, although differences in sample collection (umbilical cord vs venipuncture) could also explain these differences. A future study controlling for differences in sampling methods would be useful to further explore this observation.

**Extended Data Fig. 7:**
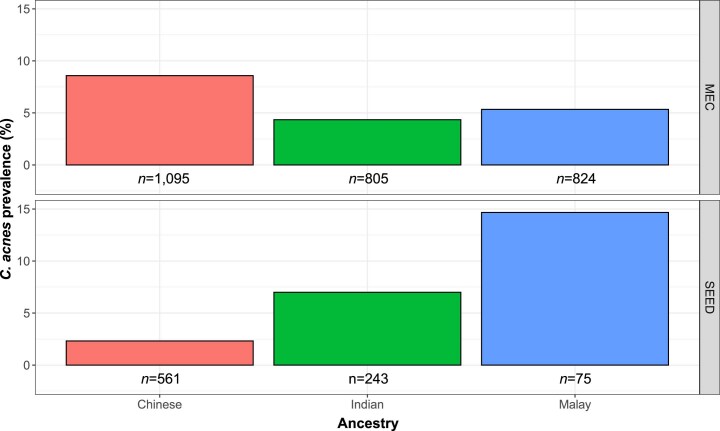
Inconsistent associations between *C. acnes* and genetic ancestry. Samples were stratified by source cohort and genetic ancestry to calculate *C. acnes* prevalence. Only the cohorts where a significant (p < 0.05) association between the presence of *C. acnes* and genetic ancestry was found are shown (*that is*, MEC and SEED). The number of samples used as the denominator when calculating prevalence is annotated. Source data

Next, we tested for pairwise associations between eight host phenotypes that were documented on the day of blood collection and the presence of each of the 117 blood microbial species. These host phenotypes were: sex, ancestry, age, body mass index (BMI), blood total cholesterol (TC), blood triglycerides (TG), systolic and diastolic blood pressure (SBP and DBP). True associations are expected to be consistent across cohorts in our dataset since they were sampled from the same population. We found only five significant microbe–phenotype associations (two-sided Fisher’s exact or Mann-Whitney *U* test, *P* < 0.05; Supplementary Table 3) after adjusting for multiple comparisons. Notably, all but one of the significant associations were present in only one cohort. The exception was *C. acnes*, which was more prevalent in individuals of Malay ancestry within the SEED cohort, but more prevalent in Chinese individuals within the MEC cohort (Extended Data Fig. 7). These cohort-specific differences could be due to other demographic variables that were not recorded in this study, or perhaps from *C. acnes* subspecies differences. To ensure that we did not miss associations due to the possible nonlinearity of host phenotype and microbial relationships, we derived categorical phenotypes. These included being elderly (age ≥ 65) and other measures of ‘poorer health’, such as being obese (BMI > 30), having high blood triglycerides (TG > 2.3 mmol l^−1^), total cholesterol (TC ≥ 6.3 mmol l^−1^) or blood pressure (SBP ≥ 130 and DBP ≥ 80). We found no significant associations between these derived phenotypes and the presence of any microbial species (two-sided Fisher’s exact test, *P* > 0.05; Supplementary Table 4). Collectively, these results suggest no consistent associations between microbial presence in blood and the host phenotypes tested within a healthy population.

## Discussion

We present presumably the largest-scale analysis so far of microbial signatures in human blood while accounting for computational and contamination artefacts and found no evidence for a common blood microbiome across healthy individuals. Instead, we observed sporadic instances of blood harbouring DNA from single microbial species of diverse bodily origins, some of which might be actively replicating. The bloodstream allows microbes to move between different body sites in healthy individuals. However, the low prevalence of the detected species suggests that this movement is likely to be infrequent and transient. Unresolved questions remain about how interconnected the microbiomes at various body sites are, and whether these processes are altered during disease. Can perturbations to the microbial community at one body site affect those at another site? How does the host immune system asymptomatically regulate microbial presence in blood? Our study lays the groundwork for future investigations into these questions.

We employed a series of decontamination filters to differentiate microbial signatures in blood from artefactual signals associated with reagent and handling contamination, on the basis that the latter display strong batch-specific biases (Extended Data Fig. 2 and Methods). Although our approach substantially improved the signal-to-noise ratio (Fig. 1b–d), it is probably not fully effective in removing contaminants because a fraction of the 117 microbial species remaining after decontamination were still flagged as being of environmental or non-human origin (Fig. 1b,d). Future studies should leverage our comparisons to various microbial databases (Fig. 1b–d) to prioritize some of these 117 species for validation, primarily those that are not common contaminants, are detected in blood cultures and are human associated (Supplementary Table 2). Nevertheless, we could not detect a common blood microbiome despite the likely presence of residual contamination artefacts.

We observed DNA signatures of replicating microbes in blood via replication rate analyses. However, we could not distinguish signals arising from replicating microbes in blood from those derived from microbial cells that were recently replicating at other body sites before entering the bloodstream. Notably, while we detected replication signatures in 11 out of 20 species with sufficient coverage across their genomes, we could not detect any among the 20 most prevalent contaminant species identified by our decontamination filters, including species from the genera *Alcaligenes*, *Caulobacter*, *Bradyrhizobium* and *Sphingomonas*. This further indicates that the microbial species with detectable replication signatures in our dataset are not likely to be part of the ‘kitome’. These findings highlight the use of replication analyses for discriminating between putatively genuine taxa versus ‘kitome’ contaminants in future metagenomic studies.

We found no core species in human blood on the basis of low prevalence across individuals in our dataset, but this is contingent on the sensitivity of detecting microbes through sequencing. However, previous studies have shown that metagenomic sequencing is highly sensitive for the detection of blood microbes at 20–30 million reads per sample^35,54,55^. In comparison, our libraries were sequenced deeply (median = 373 million reads), suggesting that our methods do not lack sensitivity. Our prevalence estimates are also affected by the abundance thresholds used to determine whether a species is present in a single sample (Fig. 1a). These included both absolute read count and relative abundance thresholds that were defined following simulation experiments (see Methods). However, even when using a single and more relaxed relative abundance threshold of 0.001, none of the species had more than 52% prevalence (Supplementary Table 5). Furthermore, the 20 most prevalent species at this threshold are all environmental microbes, mostly comprising *Sphingomonas* and *Bradyrhizobium* species, which are common sequencing-associated contaminants^19^. Therefore, independent of our decontamination thresholds, none of the species detected qualify as core members.

We could not detect any strong co-occurrence (cooperative) or mutual exclusion (competitive) associations^56^ between species regardless of whether decontamination filters were applied. Within a microbial community, metabolic dependencies of species and metabolic complementation are key drivers of microbial co-occurrence^57^. Conversely, competitive behaviours such as nutrient sequestration and selective adhesion^58^ can lead to microbial mutual exclusion. The lack of strong associations between microbial species points to the absence of an interacting microbial community in the blood of healthy humans. Of note, since our dataset was derived from circulating venous blood, we were unable to measure microbial interactions that may be occurring at other sites of the bloodstream, such as the inner endothelial lining. Experiments investigating bacterial adhesion to endothelial linings may provide further insights as to whether such interactions exist.

The availability of blood culture records from the same country of origin as our blood samples enabled a reliable comparison of microbial prevalence in the healthy population and in the clinic^59^. Some of the variation in prevalence estimates may be due to differences in detection methods. However, previous studies have shown a strong concordance between culture and sequencing-based detection^35,54,60,61^, indicating that most of the observed variation is not due to the use of different detection methods. Our results indicate that microbial presence in blood does not always lead to disease. This is consistent with our other observation that microbial DNA detected in healthy asymptomatic individuals tends to be from commensals, which may inherently be less virulent and better tolerated by the host compared to disease-causing pathogens. Indeed, circulating commensals may exhibit immunomodulatory phenotypes, akin to gut microbes^62,63^, facilitating asymptomatic co-existence with the host. Perhaps, the presence (or lack) of immunomodulatory properties may determine whether an individual with bacteraemia is asymptomatic or septic. Further exploration of the immunomodulatory activities of commensals vis-à-vis common blood culture pathogens may aid the design of therapies that modulate dysregulated host responses during sepsis^1^.

We found no consistent associations between both measured (for example, TC, SBP) or derived (for example, obesity) host phenotypes, and microbial presence. This suggests that the risk of transient microbial translocation across our cohorts of healthy adults is consistent across host phenotypes. However, this may not hold for diseased individuals since microbial DNA profiles in blood have been used to delineate health versus disease states, such as sepsis^35,54,55,60,61,64^ and a range of other diseases unrelated to bloodstream infections^30,52,65^. These studies highlight the promise of blood metagenomic sequencing for developing diagnostic, prognostic or therapeutic tools, but the biological basis of their findings remain unclear. One hypothesis is that mucosal and epithelial barrier integrity is compromised during disease or physiological stress^66^, leading to higher translocation rates of microbes into the bloodstream and resulting in altered blood microbial profiles. Future studies testing this hypothesis may consider a focus on the gut or mouth-associated bacteria that were detected in our study (for example, *Bifidobacterium adolescentis, Faecalibacterium prausnitzii, Streptococcus mitis*). Further investigations into these mechanisms may improve our understanding of why blood microbial profiles correlate with health status, and our characterization of the diversity of species in the blood of healthy individuals forms a crucial baseline to do so.

**Extended Data Fig. 8:**
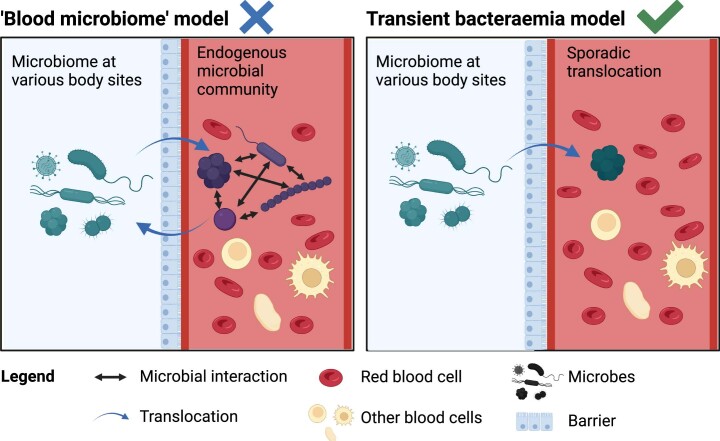
Potential models for microbes in blood. Our findings suggest that there is no consistent circulating blood microbiome (that is, the blood microbiome model). The more likely model is where microbes from other body sites transiently and sporadically translocate into blood. Created with BioRender.com under an academic subscription. Source data

In conclusion, if we take the definition of a ‘microbiome’ as a microbial community whose member species interact among themselves and with their ecological niche^9^, we found no consistent circulating blood microbiome in healthy individuals (Extended Data Fig. 8). Sporadic and transient translocation of commensals from other body sites into the bloodstream is the more parsimonious explanation for the observation that most blood microbes are commensals found in other body sites. Furthermore, the relatively low prevalence of microbes in blood suggests rapid clearance of translocated microbes rather than prolonged colonization. On the basis of these findings, we advocate against the use of the terms ‘blood microbiome’ or ‘circulating microbiome’, which are potentially misleading when referring to the detection of microbial DNA or of microbial cells in blood due to transient translocation events.

## Methods

### Datasets

All individuals in the participating cohorts were recruited with signed informed consent from the participating individual or parent/guardian in the case of minors. All cohort studies were approved by relevant institutional ethics review boards and a summary of the cohort demographics and the ethics review approval reference numbers are provided in Supplementary Table 1. Our sequencing dataset, also known as the SG10K_Health dataset (https://www.npm.sg/collaborate/partners/sg10k/), comprises shotgun sequencing libraries of DNA extracted from the whole blood or umbilical cord blood of 9,770 healthy Singaporean individuals who were recruited as part of six independent cohorts. Individuals were deemed to be healthy if they did not have any personal history of major disorders such as stroke, cardiovascular diseases, cancer, diabetes and renal failure. Oral health information was not collected and therefore was not part of the exclusion criteria. Whole blood for sequencing was collected via venipuncture only from the five adult cohorts (median age = 49; interquartile range = 16): Health for Life in Singapore (HELIOS, *n* = 2,286), SingHealth Duke-NUS Institute of Precision Medicine (PRISM, *n* = 1,257), Tan Tock Seng Hospital Personalised Medicine Normal Controls (TTSH, *n* = 920), Singapore Epidemiology of Eye Diseases (SEED, *n* = 1,436)^67,68^ and the Multi-Ethnic Cohort (MEC, *n* = 2,902)^69^. Additionally, cord blood was collected only for the birth cohort Growing Up in Singapore Towards healthy Outcomes (GUSTO, *n* = 969)^70^. Measurement of host phenotypes was performed on the day of blood collection, except for the GUSTO cohort where measurements were taken at a later timepoint when the children were at a median age of 6.1 years (interquartile range = 0.1). Individuals were broadly categorized, in a previous study^71^, into four ethnic categories representing distinct genetic ancestries: Chinese (59%), Malays (19%), Indians (21%) and Others (1%). All individuals were deemed healthy at the point of recruitment if they did not include any self-reported diseases in the recruitment questionnaires. No participant compensation was provided within the context of this study. No statistical methods were used to pre-determine sample sizes but our sample sizes far exceed those reported in previous publications (reviewed in ref. ^8^).

Additionally, we retrieved anonymized blood culture records from Singapore General Hospital, the largest tertiary hospital in Singapore. These records spanned the years 2011–2021 and included aerobic, anaerobic and fungal blood cultures taken from 282,576 unique patients. These blood cultures were ordered as part of routine clinical management, that is, when clinically indicated for the investigation of bacteraemia or fungemia. Blood cultures were performed and analysed following hospital standard operating procedures. In brief, blood samples were collected aseptically and inoculated into BD BACTEC bottles at the bedside (BD BACTEC Plus Aerobic/F culture vials plastic (442023) for aerobic blood culture, BD BACTEC Plus Anaerobic/F culture vials plastic (442022) for anaerobic blood culture and Myco/F Lytic (42288) for fungal blood culture). The inoculated bottles were transported to the diagnostic laboratory at ambient temperature and incubated in the BD BACTEC FX blood culture system on arrival. Aerobic and anaerobic blood culture bottles were incubated for a maximum of 5 d, and fungal blood culture bottles were incubated for a maximum of 28 d. Blood culture bottles that were flagged positive by the BD BACTEC FX blood culture system were inoculated onto solid media, and the resultant colonies were identified using a combination of biochemical tests and matrix assisted laser desorption ionization-time of flight mass spectrometry (MALDI-TOF MS) (Bruker microflex LRF).

### Sample preparation and batch metadata

Samples were processed in batches and were not randomized for sequencing. However, batch information for each sample was retained and used to correct for batch-specific effects. This includes the type of extraction kits and library preparation kits used, and lot numbers for the SBS kits, PE Cluster kits and sequencing flowcells used. DNA from whole blood was extracted using one of six different DNA extraction kits. Paired-end 151 bp sequencing with an insert size of 350 bp was performed for up to 15-fold or 30-fold coverage of the human genome. Library preparation was performed using one of three library preparation kits. Sequencing was performed on the Illumina HiSeq X platform with HiSeq PE Cluster kits and HiSeq SBS kits. All reagent kits used, the number of batches and the number of samples processed per batch are provided in Supplementary Table 6.

### Data pre-processing and quality control

The bioinformatic processing steps applied to the sequencing libraries are summarized in Fig. 1a. Read alignment of sequencing reads to the GRCh38 human reference genome was performed as described in a separate study^72^ using BWA-MEM v0.7.17^73^. We retrieved read pairs where both members of the pair did not map to the human genome using Samtools v1.15.1^74^ and Bedtools v2.30.0^75^, after which we performed quality control of the sequencing reads. We trimmed low-quality bases at the ends of reads with quality <Q10 (base-quality trimming) and discarded reads with average read quality less than Q10 (read-quality filter). We also discarded low-complexity sequences with an average entropy less than 0.6, with a sliding window of 50 and *k*-mer length of 5 (low-complexity read filter). All basic quality control steps were performed using bbduk from the BBTools suite v37.62 (sourceforge.net/projects/bbmap/).

### Taxonomic classification of blood sequencing libraries

Taxonomic classification of non-human reads was done using Kraken2 v2.1.2^23^ with the ‘–paired’ flag. We used the PlusPF database (17 May 2021 release; https://genome-idx.s3.amazonaws.com/kraken/k2_pluspf_20210517.tar.gz), which includes archaeal, bacterial, viral, protozoan and fungal references. Of all non-human read pairs, 72% were classified as microbial at the species level, yielding 8,890 species. Samples with fewer than 100 microbial read pairs were removed, resulting in a final dataset comprising 8,892 samples, with a median microbial read-pair count of 6,187.

**Extended Data Fig. 9:**
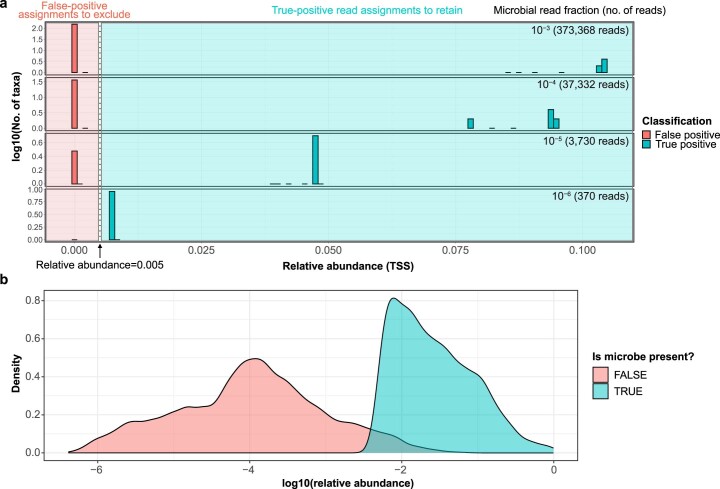
Simulation experiments to determine the abundance cutoff for reducing false-positive species assignments. (a) Histogram of the relative abundance of true-positive and true-negative Kraken2 species assignments. Approximately 373 million total reads were generated from human (GRCh38) and 10 microbial reference genomes at various microbial read fractions using *InSillicoSeq*. An abundance cutoff delineating the false-positive (FP) from true was selected (relative abundance=0.005) that retains and excludes all true-positive and false-positive Kraken2 species assignments, respectively. (b) Relative abundance distributions of taxa considered present or absent as demarcated by our abundance thresholds (that is, relative abundance ≤0.005, read pairs assigned ≤10). Source data

To minimize noise in the taxonomic assignments, we defined a set of abundance thresholds whereby species with abundance values less than or equal to these thresholds (that is, relative abundance ≤0.005, read pairs assigned ≤10) were counted as absent (set to zero read counts). We performed simulations to systematically determine a relative abundance threshold that minimizes false-positive species assignments. Sequencing reads were simulated using InSilicoSeq v1.5.4^76^, with error models trained on the SG10K_Health sequencing libraries and processed using the same bioinformatic steps as the SG10K_Health dataset to obtain microbial taxonomic profiles. We simulated 373 million reads equivalent to the median library read count of all samples, comprising reads from the GRCh38 human reference and ten microbial genomes (*Yersinia enterocolitica*, *Leclercia adecarboxylata*, *Moraxella osloensis*, *Streptococcus pneumoniae*, *Pasteurella multocida*, *Staphylococcus epidermidis*, *Actinomyces viscosus*, *Torque teno virus*, *Human betaherpesvirus 6A* and *Candida albicans*) in various proportions. Due to read misclassification, some of the simulated reads were erroneously assigned to another species and produced false positives. A final relative abundance threshold of 0.005 that delineated these false-positive assignments from true positives was selected (Extended Data Fig. 9a). Following the application of these thresholds, the relative abundance distribution of microbial taxa classified as present was found to be distinct from the distribution of those classified as absent (Extended Data Fig. 9b). Furthermore, the distribution of abundances for microbe-negative samples is centred around a relative abundance of 0.0001, that is, at least tenfold below the typical relative abundance thresholds used to determine whether a taxon is present or absent (0.001–0.045^14^). Relative abundances were calculated by dividing the species-specific microbial read count in a sample by the total number of microbial reads assigned to that sample.

### Decontamination filters

**Extended Data Fig. 10:**
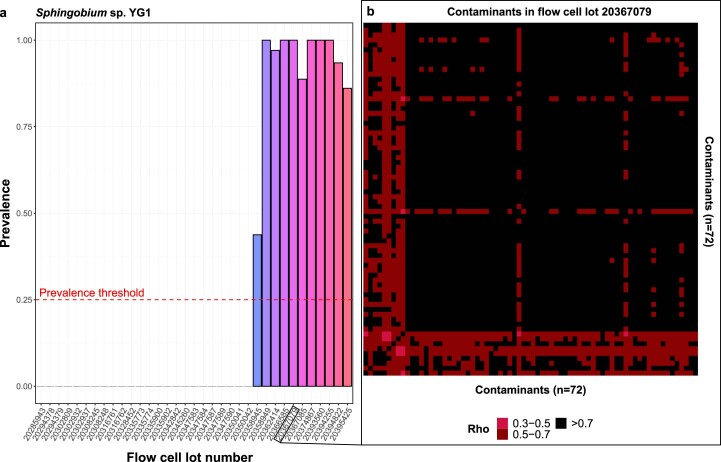
Illustration of decontamination filters used. (a) The prevalence filter flagged *Sphingobium sp. YG1* as a contaminant because its prevalence in at least one batch (that is, flow cell lot used) is greater than 25% (threshold indicated by dotted red line) and more than two-fold higher than the prevalence in at least one other batch. (b) Heatmap of pairwise Spearman’s Rho (that is, correlation) between the 72 contaminant species identified by the correlation filter for flow cell batch 20367079. The highly correlated nature of these species indicates that they are indeed likely contaminants specific to batch 20367079. Source data

After application of the presence/absence filter, we identified and removed putative contaminants using established decontamination heuristics^26^ that have been validated in previous studies^27,28^, before our downstream analyses. These rules were applied using eight types of batch information: source cohort, DNA extraction kit type, library preparation kit type, lot numbers for sequencing-by-synthesis kit (box 1, box 2), paired-end cluster kit (box 1, box 2) and sequencing flow cell used. Other batch information such as the pipettes and consumables used, or storage location and duration were not recorded but could potentially contribute to some level of batch-specific contamination. However, these batches are expected to be correlated with the other types of batch information available, hence the resultant contaminants could in theory be accounted for using our filters. We describe the four decontamination filters used, as shown in Fig. 1a, in sequential order:

1. Prevalence filter. A microbial species is considered a contaminant specific to a batch if it is present at greater than 25% prevalence in that batch and has greater than a twofold higher prevalence than that for any other batch. Batches with fewer than 100 samples were excluded from this analysis. This filter is based on the principle that species which are highly prevalent in some batches but lowly prevalent or absent in others are likely contaminants^26^. We illustrate this for an example species in Extended Data Fig. 10a.
2. Correlation filter. A microbial species is considered a contaminant if it is highly correlated (Spearman’s *ρ* > 0.7) with any contaminant within the same batch, as identified by the prevalence filter. This filter is based on the principle that contaminants are highly correlated within the same batch^26^. Spearman’s *ρ* was calculated using centred log-ratio-transformed^77^ microbial relative abundances. Centred log-ratio transformations and Spearman’s *ρ* were calculated using the clr function of the compositions package^78^ and cor.test function in R. We illustrate this within-batch correlation for an example species in Extended Data Fig. 10b.
3. Batch filter. A non-contaminant microbial species must be detected in samples processed by at least two reagent kit batches or reagent types. That is, any species that is only detected in a single batch for any of the reagent kits used (Supplementary Table 6) are considered contaminants. This filter is based on the principle that species that can be repeatedly observed across different reagent batches are more likely to reflect genuine non-contaminant signals^26^. Library preparation kit type was excluded from this analysis since only three kit types were used, with 86% of samples processed using one of the kits.
4. Read-count filter. A microbial species is considered a sequencing or analysis artefact if it is not assigned at least 100 reads in at least one sample. This filter is based on the principle that species that are always assigned a low number of read pairs never exceeding the background noise within sequencing libraries are more likely to be artefactual rather than genuine signals. An example of an artefactual species is ‘*Candidatus* Nitrosocosmicus franklandus’, which was assigned at most 22 read pairs by Kraken2 across 21 sequenced samples.

To demonstrate the effectiveness of our decontamination filters, we additionally tested our results against the null hypothesis that the 117 microbial species retained after decontamination produced the same proportions of species classified as likely contaminants, human-associated or detected in blood culture compared to picking these species at random. In this analysis, we generated 1,000 sets of 117 microbial species that were randomly selected from the list of species before decontamination and compared the species to the three databases (see Fig. 1b–d). *P* values were calculated by taking the proportion of random iterations that generated proportions of species classified as likely contaminants, detected in blood or human-associated that were as extreme or more extreme than those observed for the 117 species retained by our decontamination filters.

### Characterization of microbial species

We classified microbial species as human-associated or not on the basis of a published host–pathogen association database^31^. In this database, host–pathogen associations are defined by the presence of at least one documented infection of the host by the pathogen^31^. For species that were not found in this database, we performed a systematic PubMed search using the search terms: (microbial species name) AND (human) AND ((infection) OR (commensal)). Similarly, species that had at least one published report of human colonization/infection were considered human-associated. Additionally, we classified the potential body site origins for each microbial species using the Disbiome database, which collects data and metadata of published microbiome studies in a standardized way^34^. We extracted the information for all microbiome experiments in the database using the URL: ‘https://disbiome.ugent.be:8080/experiment’ (accessed 26 April 2022). We first extracted microbe-to-sample-type mappings from this information (for example, *C. acnes*→skin swab). We then manually classified each sample type into different body sites (for example, skin swab→skin). This allowed us to generate microbe-to-body-site mappings. Sample types with ambiguous body site origins (for example, abscess pus) were excluded. The range of sample types within the Disbiome database used to derive the microbe-body-site mappings are provided in Supplementary Table 7. Finally, we classified microbial species on the basis of their growth requirements, with reference to a clinical microbiology textbook^79^. Viruses were classified as obligate intracellular. The microbiological classifications for each species are provided in Supplementary Table 2.

### Estimating coverage breadth and bacterial replication rates

We performed read alignment of sequencing libraries to microbial reference genomes using Bowtie v2.4.5^80^ with default parameters. In total, we used references for 28 of the 117 microbial species detected in blood, comprising all bacterial species with at least 1,000 Kraken2-assigned read pairs in a single sample and all viral species (*n* = 5). For each species, we aligned the microbial reads of five sample libraries with the most reads assigned to that species, to the reference genome of that species. For each sample and microbial genome, the genome coverage per position was computed using the pileup function of the Rsamtools v2.8.0 package^81^ in R. In principle, the recovery of a large fraction of a microbial genome, as opposed to sporadic reads mapping to particular regions on said genome, provides a higher confidence that the species is truly present in a sample^24,25^. We could recover at least 10% of the microbial genomes for 27 of the 28 species (96%). Since it is difficult to assess coverage breadth for a species covered by a low number of reads, we only performed this analysis on all viruses (*n* = 5) and all bacterial species with at least 1,000 Kraken2-assigned read pairs (*n* = 23), which corresponds to ~10% coverage over a typical 3 Mbp bacterial genome (assuming non-overlapping reads). For the replication rate analyses, PTR values were calculated using the bPTR function in iRep v1.1.0^21^, which is based on a previously proposed method^22^. The *Ori* and *Ter* positions were determined on the basis of the coverage peaks and troughs (Fig. 3, in red and blue, respectively). *Ori* and *Ter* positions were also calculated using a cumulative GC-skew line, which is expected to be in anti-phase with the sinusoidal coverage pattern across the genome^39^ (Fig. 3, in green).

### Microbial networks

Microbial co-occurrence/mutual exclusion associations were computed using the SparCC algorithm^50^ implemented in the SPIEC-EASI v1.1.2 package^82^ in R, and the microbial networks were visualized using Igraph v1.2.9^83^. We excluded the birth cohort GUSTO since it is of a different demographic that may possess a distinct set of microbial associations.

### Detecting associations between microbial taxonomic profiles and host phenotypes

We tested for microbe–host phenotype associations within individual cohorts separately. For the two categorical host phenotypes, genetic sex and ancestry, we tested for differences in the prevalence of each microbial species between the different categories using a two-sided Fisher’s exact test (fisher.test function in R). For the continuous variables (age, BMI, TC, TG, SBP and DBP), we used a two-sided Mann-Whitney U test (wilcox.test function in R) to test for differences in the distributions of the variables when a species was present or absent. Benjamini-Hochberg multiple-testing correction was applied only after consolidating the *P* values from both tests and for all cohorts using the P.adjust function in R. Statistical tests were only performed if a species was present in at least 50 samples in total. Separately, for derived phenotypes (that is, being elderly or measures of ‘poorer health’), we used the Fisher’s exact test before applying Benjamini-Hochberg multiple-testing correction. In all cases, samples with missing host phenotype data were excluded. All data analysed fulfilled the assumptions of the statistical tests used.

### Data analysis and visualization

All data analyses were performed using R v4.1.0 or Python v3.9.12. Visualizations were performed using ggplot v3.3.5^84^. Extended Data Fig. 8 was created using BioRender.com under an academic subscription.

### Reporting summary

Further information on research design is available in the Nature Portfolio Reporting Summary linked to this article.

## Supplementary information

Reporting Summary

Supplementary Tables 1–9. Supplementary Table 1. Demographics of the 9,970 healthy individuals in our dataset from six independent cohorts. The approval reference numbers from the relevant institutional ethics review boards are provided. Table 2. Final list of 117 microbial species that were detected in the blood samples of 8,892 individuals following stringent decontamination. Table 3. Summary of the five significant microbe–phenotype associations (adjusted *P* < 0.05) after adjusting for multiple comparisons using the Benjamini-Hochberg procedure. Mann-Whitney *U* statistics are provided or left blank where a two-sided Mann-Whitney *U* test or Fisher’s exact test were used, respectively. Table 4. Summary of all associations between microbial presence (that is, presence of any microbial species) and derived host phenotypes. We performed two-sided Fisher’s exact tests to assess the statistical significance of these associations. Tests were performed for each cohort separately. Benjamini-Hochberg procedure was applied to correct for multiple-testing. Table 5. Prevalence of all microbial species detected after applying a relaxed abundance filter where species were considered to be present if their relative abundance was greater than 0.001, but before decontamination was performed. Prevalence was calculated as the percentage of samples (*n* = 8,892) where the particular microbial species was detected. Table 6. Metadata of the types or lot numbers of reagent kits used for each sample (*n* = 9,770). Table 7. The range of sample types and the microbial species associated with them as retrieved from the Disbiome database. Each sample type was assigned an originating body site to derive the microbe-to-body-site mappings used in our analyses. Sample types with ambiguous body site origins (for example, abscess pus) were excluded. Table 8. List of microbial reference genomes used in our study and their corresponding NCBI accession numbers. The types of analysis for which the reference genomes were used are annotated. Table 9. List of members of the SG10K_Health Consortium and their affiliations.

## Data Availability

The authors of this study do not own the rights to the SG10K_Health dataset, and this dataset is under controlled access to ensure good data governance, responsible data use, and that the dataset is only used for the intended research purposes in compliance with SG10K_Health study cohort IRB and ethics approval. Users interested in accessing the SG10K_Health individual-level data (WGS and VCF files) are required to submit a Data Access Request outlining the proposed research for approval by the NPM Data Access Committee (DAC), which convenes monthly. The forms and data access policy can be downloaded via the SG10K_Health portal (https://npm.a-star.edu.sg/help/NPM) upon registration with an institutional email address. For more information, users can contact the National Precision Medicine Programme Coordinating Office, A*STAR (contact_npco@gis.a-star.edu.sg). The average turnaround timeframe for a request is 4–6 weeks from receipt of request to receiving a notification outcome from the NPM DAC on whether the application is accepted/rejected/requires amendments. The approved requestor will be asked to sign a non-negotiable data access agreement to ensure that (1) the data are used only for the proposed research purpose, (2) no attempt is made to re-identify the participants, (3) there is no onward sharing of the data to a third party and (4) a standard acknowledgement statement is included in the manuscript. All source data used for our analyses are hosted on Zenodo (10.5281/zenodo.7665281), including Kraken2 taxonomic profiles of all real and simulated sequencing libraries, and the anonymized blood culture records. The accession numbers for all genome references used are provided in Supplementary Table 8. The PlusPF database (17 May 2021 release) can be accessed online (https://genome-idx.s3.amazonaws.com/kraken/k2_pluspf_20210517.tar.gz). The Disbiome database^34^ can be accessed online (https://disbiome.ugent.be:8080/experiment). The host–pathogen database^31^ can be accessed through FigShare (10.6084/m9.figshare.8262779). Source data are provided with this paper.

## Source data

Source Data Figs. 1–4, Extended Data Figs. 1–10 Statistical source data.

## Code availability

All custom codes used to perform the analyses reported here are hosted on GitHub (https://github.com/cednotsed/blood_microbial_signatures.git).
