# Supplementary material for: No evidence for a common blood microbiome based on a population study of 9,770 healthy humans
Source: Nat Microbiol. 2023 Mar 30;8(5):973–85. doi: 10.1038/s41564-023-01350-w (PMC10159858; doi:10.1038/s41564-023-01350-w)
Supplement: Supplementary file 1 — Reporting Summary [file 41564_2023_1350_MOESM1_ESM.pdf]

nature portfolio

Corresponding author(s): Cedric C.S. Tan

Last updated by author(s): Feb 15, 2023

Reporting Summary

Nature Portfolio wishes to improve the reproducibility of the work that we publish. This form provides structure for consistency and transparency in reporting. For further information on Nature Portfolio policies, see our Editorial Policies and the Editorial Policy Checklist.

Statistics

For all statistical analyses, confirm that the following items are present in the figure legend, table legend, main text, or Methods section.

n/a	Confirmed	
☐	☒	The exact sample size (*n*) for each experimental group/condition, given as a discrete number and unit of measurement
☒	☐	A statement on whether measurements were taken from distinct samples or whether the same sample was measured repeatedly
☐	☒	The statistical test(s) used AND whether they are one- or two-sided<br>*Only common tests should be described solely by name; describe more complex techniques in the Methods section.*
☐	☒	A description of all covariates tested
☐	☒	A description of any assumptions or corrections, such as tests of normality and adjustment for multiple comparisons
☐	☒	A full description of the statistical parameters including central tendency (e.g. means) or other basic estimates (e.g. regression coefficient) AND variation (e.g. standard deviation) or associated estimates of uncertainty (e.g. confidence intervals)
☐	☒	For null hypothesis testing, the test statistic (e.g. *F*, *t*, *r*) with confidence intervals, effect sizes, degrees of freedom and *P* value noted<br>*Give P values as exact values whenever suitable.*
☒	☐	For Bayesian analysis, information on the choice of priors and Markov chain Monte Carlo settings
☒	☐	For hierarchical and complex designs, identification of the appropriate level for tests and full reporting of outcomes
☒	☐	Estimates of effect sizes (e.g. Cohen's *d*, Pearson's *r*), indicating how they were calculated

Our web collection on statistics for biologists contains articles on many of the points above.

Software and code

Policy information about availability of computer code

Data collection	No software was used for data collection.

Data analysis

R 4.1.0
Python 3.9.12
Samtools 1.15.1
Kraken 2.1.2
Insilicoseq 1.5.4
bwa 0.7.17
bbtools 37.62
bedtools 2.30.0
blast 2.5.0
bowtie2 2.4.5
irep 1.1.0
Igraph 1.2.9
SpiecEasi 1.1.2
Rsamtools 2.8.0
compositions 2.0.2
ggplot 3.3.5

All custom code used to perform the analyses reported here are hosted on GitHub (https://github.com/cednotsed/blood_microbial_signatures.git).

For manuscripts utilizing custom algorithms or software that are central to the research but not yet described in published literature, software must be made available to editors and reviewers. We strongly encourage code deposition in a community repository (e.g. GitHub). See the Nature Portfolio guidelines for submitting code & software for further information.

Data

Policy information about availability of data

All manuscripts must include a data availability statement. This statement should provide the following information, where applicable:

- Accession codes, unique identifiers, or web links for publicly available datasets
- A description of any restrictions on data availability
- For clinical datasets or third party data, please ensure that the statement adheres to our policy

The dataset is under controlled access to ensure good data governance, responsible data use, and that the dataset is only used for the intended research purposes in compliance with SG10K_Health study cohort IRB and ethics approval. Users interested in accessing the SG10K_Health individual-level data (WGS and VCF files) are required to submit a Data Access Request outlining the proposed research for approval by the NPM Data Access Committee (DAC), which convenes monthly. The forms and data access policy can be downloaded via the SG10K_Health portal (https://npm.a-star.edu.sg/help/NPM). For more information, users can contact the National Precision Medicine Programme Coordinating Office, A*STAR (contact_npco@gis.a-star.edu.sg). The average turnaround timeframe for a request is 4-6 weeks from receipt of request to receiving a notification outcome from the NPM DAC on whether the application is accepted/rejected/requires amendments. The approved requestor will be asked to sign a non-negotiable data access agreement to ensure the data is used only for (1) the proposed research purpose, (2) no attempt to re-identify the subjects, (3) no onward sharing of the data to a third party, and (4) to include a standard acknowledgement statement in the manuscript. All source data used for our analyses are hosted on Zenodo (https://doi.org/10.5281/zenodo.7368262), including Kraken2 taxonomic profiles of all real and simulated sequencing libraries, and the anonymised blood culture records. The accession numbers for all genome references used are provided in Supplementary Table 8. The PlusPF database (17th May 2021 release) can be accessed online (https://genome-idx.s3.amazonaws.com/kraken/k2_pluspf_20210517.tar.gz). The Disbiome database34 can be accessed online (https://disbiome.ugent.be:8080/experiment). The host-pathogen database31 can be accessed through FigShare (https://doi.org/10.6084/m9.figshare.8262779).

Human research participants

Policy information about studies involving human research participants and Sex and Gender in Research.

Reporting on sex and gender

Summary of cohort demographics are provided in Supplementary Table 1. Whole blood for sequencing was collected via venipuncture only from the five adult cohorts (median age=49; interquartile range=16): Health for Life in Singapore (HELIOS; n=2,286), SingHealth Duke-NUS Institute of Precision Medicine (PRISM, n=1,257), Tan Tock Seng Hospital Personalised Medicine Normal Controls (TTSH, n=920), Singapore Epidemiology of Eye Diseases (SEED, n=1,436)[Refs 68,69], and the Multi-Ethnic Cohort (MEC, n=2,902)[Ref 70]. Additionally, cord blood was collected only for the birth cohort Growing Up in Singapore Towards healthy Outcomes (GUSTO; n=969)[Ref 71]. Measurement of host phenotypes was performed on the day of blood collection, except for the GUSTO cohort where measurements were taken at a later timepoint when the children were at a median age of 6.1 (interquartile range=0.1).

Population characteristics

All individuals recruited were deemed healthy based on self-reports. Individuals were categorised, in a previous study [Ref 72], into four ethnic categories representing distinct genetic ancestries: Chinese (59%), Malays (19%), Indians (21%) and Others (1%).

Recruitment

Individuals were deemed to be healthy if they do not have any personal history of major disorders such as stroke, cardiovascular diseases, cancer, diabetes and renal failure. Oral health information was not collected and therefore not part of the exclusion criteria. All individuals were deemed healthy at the point of recruitment if they did not include any self-reported diseases in the recruitment questionnaires.

Ethics oversight

All individuals in the participating cohorts were recruited with signed informed consent from the participating individual or parent/guardian in the case of minors. All studies were approved by relevant institutional ethics review boards and a

summary of the cohort demographics and the ethics review approval reference numbers are provided in Supplementary Table 1.

Note that full information on the approval of the study protocol must also be provided in the manuscript.

Field-specific reporting

Please select the one below that is the best fit for your research. If you are not sure, read the appropriate sections before making your selection.

☒ Life sciences ☐ Behavioural & social sciences ☐ Ecological, evolutionary & environmental sciences

For a reference copy of the document with all sections, see nature.com/documents/nr-reporting-summary-flat.pdf

Life sciences study design

All studies must disclose on these points even when the disclosure is negative.

Sample size	We used all sample data that was available (n = 9770). Sample size is large enough for all statistical analyses used.
Data exclusions	Sequencing libraries with less than 100 assigned microbial read pairs were excluded as they do not provide sufficient and meaningful microbiological information.
Replication	The data analysed in this study is cross-sectional and all sequencing libraries were generated from samples collected from independent individuals. All sequencing libraries used in this study were from distinct individuals.
Randomization	Samples were processed in batches and were not randomised for sequencing. However, batch information for each sample was retained and used to correct for batch-specific effects.
Blinding	No experimental groups were assigned to samples in this study as all samples were 'blood collected from healthy individuals' so blinding is not relevant. However, technicians involved in the processing of the samples did not have access to participant metadata.

Reporting for specific materials, systems and methods

We require information from authors about some types of materials, experimental systems and methods used in many studies. Here, indicate whether each material, system or method listed is relevant to your study. If you are not sure if a list item applies to your research, read the appropriate section before selecting a response.

Materials & experimental systems

n/a	Involved in the study	
☒	☐	Antibodies
☒	☐	Eukaryotic cell lines
☒	☐	Palaeontology and archaeology
☒	☐	Animals and other organisms
☒	☐	Clinical data
☒	☐	Dual use research of concern

Methods

n/a	Involved in the study	
☒	☐	ChIP-seq
☒	☐	Flow cytometry
☒	☐	MRI-based neuroimaging
